# Possible Transient Anterior Spinal Artery Syndrome After a Celiac Plexus Neurolytic Block

**DOI:** 10.7759/cureus.43771

**Published:** 2023-08-19

**Authors:** Tyler West, Summer Pogu, Robalee Wanderman, Oludare Olatoye

**Affiliations:** 1 Department of Anesthesiology and Perioperative Medicine, Mayo Clinic, Rochester, USA; 2 Department of Pain Medicine, Twin Cities Orthopedics, Eagan, USA

**Keywords:** chronic pain management, adverse event, procedural complication, anterior spinal artery syndrome, neurolytic celiac plexus block

## Abstract

Celiac plexus blocks have been utilized to treat chronic abdominal pain of various etiologies that are refractory to medication management. This procedure is considered relatively safe; however, one rare complication is anterior spinal artery syndrome, which can result in temporary or permanent paralysis of the lower extremities.

A 67-year-old male with a history of metastatic esophageal adenocarcinoma and chronic pain refractory to high doses of opioids presented for a celiac plexus neurolytic block. The block was performed successfully with a test block containing 2% lidocaine and 0.5% bupivacaine, after which neurolysis with alcohol was completed. The patient had a syncopal episode in the post-anesthesia care unit (PACU), which resolved with fluid resuscitation without requiring advanced cardiovascular life support (ACLS). He was then discharged. On the evening of discharge, the patient had progressive lower extremity weakness to the point where he was unable to walk even with significant assistance from a family member. He went to the emergency department where a complete spine MRI was done which did not show any spinal cord defect. His physical exam showed preserved proprioception and vibration sensation with upper motor neuron exam signs. The remainder of his sensory exam was inconsistent with both reported intact sensation to pinprick and temperature with intermittently reported hyperalgesia in his lower extremities. Over the next day of admission, his weakness slowly improved. Unfortunately, the patient developed a bowel perforation during hospitalization that was non-operable, and he passed away on hospital day five.

This patient likely had anterior spinal artery vasospasm causing temporary lower extremity weakness. Given his overall debility, his physical exam was difficult, although he had intact proprioception and vibration sensation with upper motor neuron exam findings suggestive of an anterior cord process. Vasospasm could be secondary to needle placement near the artery of Adamkiewicz, alcohol, or epinephrine.

This case emphasizes the importance of recognizing anterior spinal artery syndrome despite its rarity in patients undergoing celiac plexus neurolysis. Regardless of the rarity of various complications, it is imperative that physicians discuss potential devastating complications of procedures with patients to allow for individualized decision-making. Additionally, there should be a low threshold for overnight admission after celiac plexus neurolytic blocks in patients with severe underlying systemic disease processes.

## Introduction

Fluoroscopy-guided celiac plexus blocks are typically used to alleviate visceral upper abdominal pain that is refractory to medication management [1]. The celiac plexus receives visceral afferent sensory fibers from the distal esophagus, stomach, small bowel, pancreas, spleen, liver, biliary tract, gallbladder, adrenal glands, kidneys, and large bowel up to the splenic flexure [2]. The celiac plexus block is efficacious for pain related to cancer such as pancreatic cancer and non-cancer conditions such as chronic pancreatitis [3,4]. This procedure is considered relatively safe and minimally invasive with a low complication rate [5]. One rare and potentially devastating complication of celiac plexus blocks is anterior spinal artery syndrome, which can result in lower extremity paralysis [5]. There is no incidence rate reported for this rare complication, but there are case reports [5-8].

Anterior spinal artery syndrome occurs from occlusion or vasospasm of the anterior spinal artery resulting in ischemic spinal cord infarction and motor deficits of the lower extremities [8]. The anterior spinal artery supplies the anterior two-thirds of the spinal cord, which consists of motor, temperature, and pain pathways. The artery of Adamkiewicz is the dominant feeding vessel of the anterior spinal artery in the low thoracic and lumbar distribution. It most commonly arises from the aorta between vertebral levels T7 and L4 on the left side; however, it has been reported to arise from either side of the aorta and anywhere from T3 to L4 [9]. Since the target of a celiac plexus block is the anterior border of the L1 vertebral body, it can potentially be damaged during the celiac plexus block procedure [5]. This would result in upper motor neuron weakness of the lower extremities with intact proprioception, fine touch, and vibration sensation while having decreased or absent temperature and pain sensation. We present a case of a patient who developed transient bilateral lower extremity weakness following a neurolytic (via alcohol) celiac plexus block.

This article was previously presented as a meeting case report at the 21st Annual American Society of Regional Anesthesia and Pain Medicine (ASRA) Meeting on November 19th, 2023.

## Case presentation

**Figure 1:**
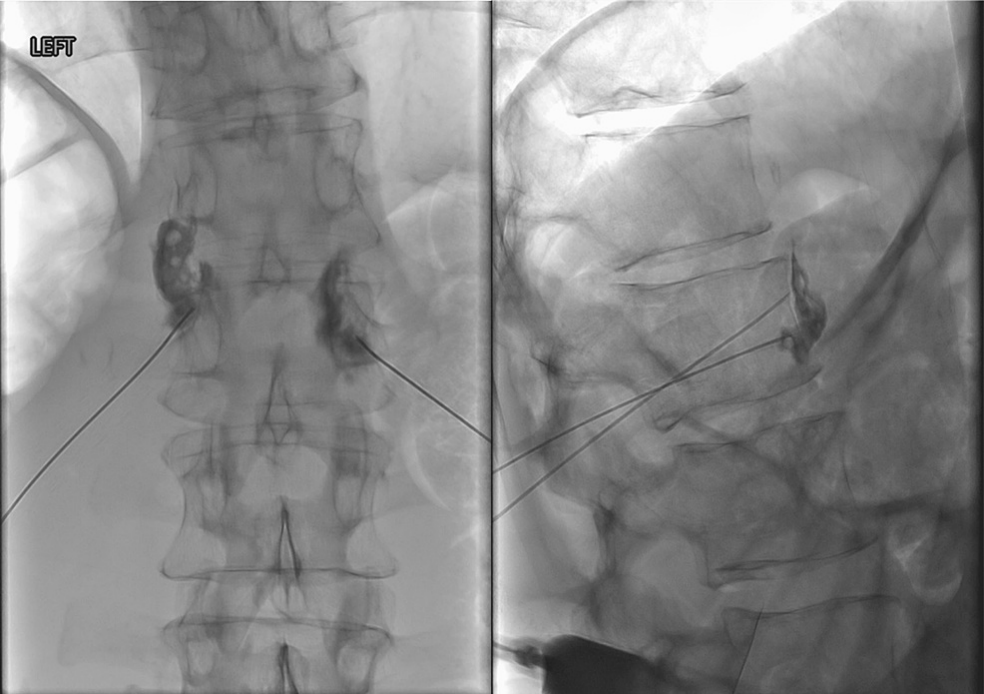
Contrast spread at anterior L1 vertebral body in AP and lateral views

The patient is a 67-year-old, 65.7 kg (BMI: 20.2) male with a history of progressive esophageal adenocarcinoma with metastasis to his pancreas. He underwent palliative chemotherapy, which was complicated by significant neuropathy in addition to multiple fractions of radiation therapy without improvement. Additionally, he was a former smoker with at least a 50-pack-year history and suffered from chemotherapy-induced cardiomyopathy with an ejection fraction of 45%-50%. His malignancy resulted in intractable visceral epigastric pain with radiation to his back. Due to the refractory nature of this pain despite being on high-dose oral opioids (hydromorphone 8 mg every three hours as needed), a fluoroscopy-guided neurolytic celiac plexus block was requested. Following a thorough clinical evaluation, the patient was deemed to be a candidate for this procedure. A pre-procedural fluid bolus of 250 mL was given through the patient’s Port-A-Cath. With the patient lying prone, two 22-gauge 5-inch spinal needles were cautiously advanced via fluoroscopy guidance using multiple views toward the anterior border of the L1 vertebral body bilaterally. This is the standard method used at Mayo Clinic. An inferior approach with a relatively steep cephalad angle was used during needle entry to guard against inadvertent pleural puncture. Of note, a pre-procedural CT of the abdomen revealed low-lying lung fields, approximately at the upper border of the L1 vertebral body. Following the final positioning of the needles at L1, iodinated contrast spread revealed standard spread with no vascular uptake, which is shown in Figure 1.

A digital subtraction angiography (DSA) was performed, which showed no vascular uptake. A test dose containing 10 mL of 2% lidocaine and 10 mL of 0.5% bupivacaine with 1:200,000 epinephrine was split equally between both needles. The test dose was negative, and no significant rise in heart rate or blood pressure was observed after 10 minutes. Additionally, the patient noticed pain relief with the test block. A sensorimotor examination was then performed, which did not show any new neurologic deficits prior to the injection of alcohol. A total of 15 mL of alcohol split between both needles was slowly injected anterior to the L1 vertebral body. There was a gradual decrease in the patient’s systolic blood pressure due to sympathectomy during the procedure; however, his mean arterial pressures were maintained above 65 mmHg with fluid administration. The procedure was completed uneventfully.

About one-hour post-procedure, the patient had a syncopal episode with a reported loss of consciousness in the postoperative area when he stood up in preparation for discharge. His blood pressure changed from 136/92 mmHg (mean arterial pressure [MAP] 107) before standing to 90/74 mmHg (MAP 79) during the syncopal episode. The patient’s baseline blood pressure was approximately 120-130/70-80 mmHg. He was placed back in his bed in Trendelenburg position, and he quickly regained full consciousness without requiring any life support measures. He received another fluid bolus with improvement in blood pressure and orthostatic symptoms. He was discharged home after significant monitoring for another hour. Prior to discharge, the patient noted significant relief in his abdominal pain. During the evening at home following the procedure, the patient developed progressive weakness without pain in his bilateral lower extremities to the point where he was not able to lift his legs against gravity while he was in bed. Additionally, he had two episodes of urinary incontinence. This prompted an urgent visit to the emergency department. On evaluation, the patient was able to passively move his legs in the horizontal plane but was still unable to lift his legs against gravity. His initial exam exhibited intact proprioception, vibration, and light touch sensation bilaterally. The remainder of his sensory exam was inconsistent. He reported intact sensation to pinprick and temperature bilaterally at times, and hyperalgesia and increased sensation to cold in his bilateral lower extremitiesat other times. He had a positive Babinski reflex and hyperreflexia in his lower extremities. Two-point discrimination was not documented. He noted that he had lower extremity weakness at baseline, requiring one- to two-hand stabilization of surrounding objects for short walks and a wheelchair for longer distances. However, he was not able to walk overnight even with significant assistance from a family member.

An initial lumbar spine MRI without contrast was performed without any spinal cord compression or signal abnormality. Neurology was consulted, after which cervical and thoracic MRIs without contrast were obtained, which did not show any evidence of an infarct or high-grade stenosis again. Over the first night of admission, the patient’s lower extremity weakness and upper motor neuron signs began to slowly improve. A repeat MRI with contrast was discussed due to the possibility of the initial imaging being too early to visualize a spinal cord infarct. Unfortunately, during the evening of hospital day two, the patient had acute worsening of his abdominal pain, melena, and anemia requiring transfusion. CT imaging found that he has pneumoperitoneum with likely bowel perforation. He was not deemed a surgical candidate and transitioned to comfort care, passing away on hospital day five. His weakness and upper motor neuron exam findings initially improved over hospital day two; however, after the bowel perforation occurred, the patient continued to have significant lower extremity paresis until passing away. Continued in-depth neurology exams were not completed after his bowel perforation as it would not have changed his management given his goals of care.

## Discussion

Herein, we describe a case of progressive lower extremity weakness following a neurolytic celiac plexus block in the setting of cancer-related abdominal pain. The exact etiology of the patient’s condition is difficult to ascertain, but anterior spinal artery syndrome is most likely. His exam findings were inconsistent; however, the timing of his lower extremity weakness in relation to the neurolytic block in addition to the patient having intact proprioception, light touch, vibration, and upper motor neuron injury signs point to anterior spinal artery syndrome. Anterior spinal artery syndrome in the setting of a celiac plexus block remains a rare but real possibility, especially in patients with severe underlying comorbidities. The patient did have baseline lower extremity weakness and edema due to his malignancy and overall debility. However, he was functional and ambulatory prior to the celiac plexus block. Given the transient nature of the patient’s symptoms and lack of objective image findings of a spinal cord infarct, he likely suffered from anterior spinal artery vasospasm rather than complete vaso-occlusion or significant ischemia. Transient vasospasm could have resulted from irritation from the needle placement near the artery of Adamkiewicz, administration of alcohol, or epinephrine injection during the procedure [8]. The patient may have been recovering from a vasospasm episode by the time he presented to the hospital, resulting in inconsistent exam findings.

Other etiologies of his lower extremity weakness considered less likely were local anesthetic or alcohol spread to the lumbar nerve roots, spinal cord metastasis, or anterior spinal artery thrombosis from hypercoagulability in the context of advanced malignancy. The patient also had episodes of hypotension, which theoretically could cause decreased perfusion of the anterior spinal cord; however, these episodes were brief. Additionally, the patient’s baseline blood pressure was normotensive. Given the patient’s MAPs were maintained greater than 65, it is less likely that his hypotensive episodes resulted in his original presentation. While hospitalized, the patient did not become hypotensive until the day of his passing, making it less likely that his persistent weakness was due to malperfusion of his spine. This was more likely due to an acutely worsening clinical condition in the context of bowel perforation.

The lack of MRI findings does not exclude vasospasm as vasospasm does not always present with MRI findings given its transient nature. While MRI is considered the most sensitive modality for the detection of tissue ischemia or infarct as mentioned previously, spinal cord infarcts may take some time to be seen on MRI after the initial insult. Therefore, the lack of MRI findings should not interrupt care if diagnostic suspicion is high. Additionally, the lack of contrast administration such as gadolinium with the spine MRI obtained during this case may have also affected the sensitivity and specificity of this diagnostic modality. It would have been interesting to see if the patient had any signs of spinal cord infarction or compression given his continued paresis after his bowel perforation; however, this did not align with his goals of care.

Although it was discussed if the pneumoperitoneum could have been caused by hypotension from the celiac plexus neurolysis, this is unlikely as he did not have prolonged cardiopulmonary effects to result in persistent generalized malperfusion. However, with his syncopal episode and overall debility, his cardiac reserve was likely decreased, and even short episodes of hypotension could result in critical end-organ ischemia. He more likely had a bowel perforation due to his overall debility and metastatic cancer, which was the suspected etiology by the trauma critical care and general surgery team. His low blood pressure was quickly treated with fluid boluses, increasing his blood pressure to a near baseline level. Additionally, his initial blood pressure on presentation to the emergency department was normotensive (131/89 mmHg), so he was unlikely persistently hypotensive at home. This emphasizes the need to adequately fluid resuscitate patients undergoing this procedure to mitigate the effects of hypotension due to the sympathectomy while also balancing resuscitation with patient comorbidities such as heart failure. Thankfully, the patient in this case had an ejection fraction that was only slightly below normal, so we were confident that he could handle the fluid resuscitation that was provided.

One aspect of this case that could have been improved is admitting the patient from the post-anesthesia care unit (PACU) rather than discharging him. After further fluid resuscitation and monitoring in the PACU, a joint decision was made between the patient and the pain medicine team that it was safe for discharge with strict return instructions. In this case, with the patient’s advanced malignancy and heart failure with reduced ejection fraction, the patient possibly would have benefited from overnight admission after his post-procedure syncopal episode. The patient would have had periodic blood pressure monitoring, further resuscitation if needed, and earlier evaluation for his lower extremity paralysis. It is difficult to postulate if an overnight admission would have changed this patient’s outcome, but this could have been a beneficial change in management.

## Conclusions

This case stresses the importance of recognizing anterior spinal artery syndrome despite its rarity in patients undergoing celiac plexus neurolysis. It also emphasizes the need for comprehensive informed consent for invasive procedures. Regardless of the rarity of various complications, physicians must discuss potential devastating complications of procedures with patients. This allows the patient to weigh the benefits and risks of an elective procedure so that the best-individualized decision is made. Lastly, a learning point to be drawn from this case is that if a patient has severe underlying systemic disease processes, there should be a low threshold for overnight hospital observation after a typical same-day discharge procedure if the patient experiences a post-procedure complication such as a syncopal episode.
